# Gut Microbiota and Liver Dysfunction in Sepsis: The Role of Inflammatory Mediators and Therapeutic Approaches

**DOI:** 10.3390/ijms252413415

**Published:** 2024-12-14

**Authors:** Aqsa Shahid, Stephen Chambers, Amy Scott-Thomas, Madhav Bhatia

**Affiliations:** Department of Pathology and Biomedical Science, University of Otago, Christchurch 8140, New Zealand; shaaq042@student.otago.ac.nz (A.S.); steve.chambers@otago.ac.nz (S.C.); amy.scott-thomas@otago.ac.nz (A.S.-T.)

**Keywords:** sepsis, gut microbiota, liver, gut–liver axis, therapeutic targets

## Abstract

Sepsis is a life-threatening complication caused by an uncontrolled immune response to infection that can lead to multi-organ dysfunction, including liver injury. Recent research has shown the critical role of gut microbiota in sepsis pathogenesis, with the gut–liver axis playing a crucial role in disease progression. Mechanisms such as the disruption of the gut barrier and liver injury pathways mediated by cytokines, chemokines, adhesion molecules, hydrogen sulfide (H_2_S). and substance P (SP) have been the focus of recent studies. Some potential biomarkers and gut microbiota-targeted therapies have shown promise as emerging tools for predicting and managing sepsis. This review describes the role of the gut–liver axis in sepsis and the potential of microbiota-targeted therapies and biomarker-driven interventions to improve sepsis outcomes.

## 1. Introduction

Sepsis is a serious, life-threatening condition associated with organ injury caused by a dysregulated host response to infection [1]. It is associated with high mortality rates worldwide, making it a global challenge. The liver is the largest organ of the body, responsible for several physiological functions such as detoxification, the production of coagulation factors, and immunity [2]. The liver plays an important role during sepsis by the regulation of the immune system through the production of several inflammatory mediators, elimination of pathogens, and regulation of metabolism. The term microbiota was first used in the early 1900s and was introduced to describe the diverse community of microbes such as viruses, bacteria, and yeast which inhabit several parts of the body, including the skin, oral cavity, lungs, and gut. The gut microbiota are a collective group of microbes that reside within the human gut [3]. In recent years, the gut microbiome has gained significant attention due to its role in health and disease. Substantial evidence has shown a close association between the gut and liver. During sepsis, disruption in the gut barrier can translocate microbes into both the liver and bloodstream, which can exacerbate inflammation. This intricate interaction between the gut and the liver can serve as a target for novel therapeutic approaches for sepsis. This review summarizes the role of the gut–liver axis in sepsis and potential therapeutic targets such as the microbiota and biomarkers to improve future sepsis management.

## 2. Overview of Sepsis

Sepsis is a serious condition characterized by a dysregulated response of the body to infection. It progresses from infection to inflammation, septic shock, and organ dysfunction [4]. In the past few years, the morbidity and mortality rates of sepsis have significantly increased, and approximately 19.7% of global deaths are caused by sepsis-associated organ failure. This increased number of deaths globally is highly alarming [5]. Several factors, such as antimicrobial resistance and an expanded use of medical interventions, alongside the broad application of immunosuppressants and chemotherapeutic agents, have contributed to this increase [6]. Furthermore, in survivors, sepsis can lead to serious complications, such as multiple organ dysfunction syndrome (MODS) [7]. The timeline from disease initiation and its subsequent progression is variable and the current standard practice for diagnosis and treatment for sepsis is limited. Conventional diagnostic methods such as microbiological culture and identification of pathogens are used to diagnose infections. Antibiotics and supportive therapy such as resuscitation fluids, vasopressors, and steroids are commonly used for the management of sepsis [8]. Several inflammatory mechanistic pathways and damage to the circulatory system contribute to the progression of sepsis and sepsis-related organ dysfunction [9]. Dysregulation of the immune system in response to infection plays a key role in the development of sepsis. Following the onset of sepsis, an excessive release of inflammatory mediators (cytokine storm) occurs to control infection. Cells of the innate immune system, such as macrophages, lymphocytes, mast cells, and epithelial cells, are activated at the site of infection. These cells help in the recruitment of circulating immune cells, such as monocytes, natural killer (NK) cells, eosinophils, platelets, neutrophils, and dendritic cells. Pathogen recognition receptors (PRRs) (present on the surface of these cells) recognize and bind to the pathogen-associated molecular patterns (PAMPs) present on bacterial cell walls. In addition, host tissue releases damage-associated molecular patterns (DAMPs), including heat shock proteins, high-mobility group box 1 protein (HMGB1), and mitochondrial DNA [10]. The binding of PRRs to PAMPs and DAMPs triggers an intracellular signaling cascade and activates transcription regulators, including activator protein 1 (AP-1) and nuclear factor-κB (NF-κB). The activation of these regulators results in the release of coagulation factors, chemokines, cytokines, and inducible nitric oxide synthetase (iNOS). Hyperactivation of these molecules contributes to the activation of the adaptive immune system and systemic inflammatory responses [11]. The immune system responds to the infection by activating pro- and anti-inflammatory pathways. After the resolution of infection, the body establishes a balance between stimulating (upregulation) and suppressing (downregulation) excessive immune responses. Furthermore, immunological memory is established to recognize and respond more effectively to future infections by the same pathogen [12]. When a primary response to infection is dysregulated, the balance between the immune system’s up- and downregulation is disrupted. As sepsis progresses, the immune system shows a hypo-inflammatory state after 24 to 48 h. During this stage, the immune system is less active, which leads to immunosuppression [1]. At this stage, patients cannot eliminate the initial infections and are more prone to develop secondary bacterial infections. Several immunosuppressive mechanisms, such as anergy, B-cell and T-cell depletion, and apoptosis, contribute to the progression of sepsis [13,14]. Several microorganisms (including Gram-positive and Gram-negative bacteria, fungi, and viruses) are involved in the pathogenesis of sepsis. The most common Gram-positive bacteria in sepsis are *Streptococcus pneumoniae* and *Staphylococcus aureus*, while among Gram-negative bacteria, *Escherichia coli*, *Klebsiella pneumoniae*, and *Pseudomonas* spp. are most common. *Candida* spp. are the most prevalent cause of fungal infections, particularly in immunocompromised patients and cancer sufferers who are undergoing chemotherapy [15]. Some viruses, such as adenoviruses, respiratory syncytial virus (RSV), parainfluenza virus, enterovirus, and influenza virus, can also cause sepsis [16,17].

## 3. The Microbiome and Its Role in the Pathogenesis of Sepsis

The gut microbiota comprise around 80% of total flora in the body and play a significant role in maintaining human health. The gut microbiome is responsible for many functions, such as defending against harmful pathogens, producing vitamins, provoking immune responses, and fermenting food [18]. The intestinal microbiota predominantly consist of six phyla, namely, *Fusobacteria*, *Bacteroidetes*, *Proteobacteria*, *Firmicutes*, *Verrucomicrobia*, and *Actinobacteria*. Among these phyla, *Bacteroidetes* and *Firmicutes* are most abundant in the gut [19], alongside fungal species such as *Saccharomyces*, *Cladosporium*, *Candida*, and *Malassezia* [20]. The gut microbiome significantly affects human health and has been called the “second genome” [7]. The gut flora plays an important role in maintaining the integrity of the intestinal barrier [21], and upon disruption, it increases intestinal permeability and causes impaired mucosal immune function, resulting in the translocation of bacteria [22]. This imbalance and loss of function can contribute to the pathogenesis of several diseases, such as diabetes, inflammatory bowel disease (IBD), obesity, and *Clostridium difficile* infection (pseudomembranous colitis) [23]. Recently, it has been reported that gut dysbiosis plays an important role in the progression of sepsis [24]. Several factors, including a disruption of epithelial barrier function, inflammation, impaired gastrointestinal motility, and hypoxia, can modulate the formation of the gut microbiota in sepsis [25,26]. Some virulent pathogens, e.g., *Trichinella spiralis* and *Vibrio cholera*, can degrade mucin, which results in the inhibition of mucus production [27], whereas *Akkermansia muciniphila* increases the production of goblet cells and restores mucus production [28]. *Lactobacillus* spp. also induce the production of mucin 2 (MUC2) and mucin 3 (MUC3), which helps in the maintenance of the mucus barrier [29,30]. Short-chain fatty acids (SCFAs, a by-product of gut bacteria) also play an important role in increased mucin production [31]. It has been found that septic patients have decreased levels of SCFAs as compared to healthy individuals and this reduction lasts for up to six weeks [32]. SCFAs also play an important role in maintaining the integrity of the gut barrier and immune responses. As the absorption of SCFAs decreases, it allows for the colonization of pathogenic bacteria and disrupts immune responses [33]. *Faecalibacterium* and *Clostridium* spp. synthesize the SCFA butyrate, which upregulates the expression of *Foxp3* (regulatory T-cell transcription factor), which promotes the differentiation of regulatory T cells [34]. It also prevents histone deacetylation, which downregulates the expression of NF-κB and results in the inhibition of the production of pro-inflammatory cytokines such as Interleukin-6 (IL-6) and Tumor Necrosis Factor-alpha (TNF-α) [35]. Recently, the results of differentially methylated region (DMR) assays revealed that several genes in colitis tissue were found to be enriched in cancer-related pathways, such as the Wnt, PI3K–AKT, MAPK, Ras, and TGF-beta signaling pathways. These findings highlight that chronic inflammation may not significantly increase the accumulation of genetic mutations but can contribute to cancer development by inducing genetic and epigenetic changes in specific genes [36,37]. When gut microbiota disruption occurs, it leads to a shift from health-promoting bacteria to pathogenic microbiota [38]. This disruption also affects the function of symbiotic bacteria, such as *Lauterella*, *Prevotella*, *Faeculus*, and *Verrucobacterium* [39]. In septic patients, the gut microbiota are less diverse and are predominantly dominated by one or a few bacterial species. It is these changes in intestinal microbial diversity which contribute to the development of sepsis [40,41]. In a prospective cohort study of more than 200 premature neonates, it was observed that a gut microbiome with higher bacterial diversity and colonization of anaerobes was associated with a lower incidence of sepsis [42]. In the absence of anaerobes, virulent bacteria such as *E. coli* and *Staphylococcus* spp. migrate into the bloodstream, resulting in sepsis [42,43]. It has been reported that microbiota dysbiosis is linked to an increase in mortality, and the intestinal microbiota can serve as a predictive marker in the prognosis of sepsis [44].

## 4. Impact of Antibiotics on Gut Microbiota

The gut microbiota can be modulated with antibiotics, commonly used to treat bacterial infections in sepsis, where antibiotics specifically targeting anaerobic bacteria significantly change microbial biodiversity. This change leads to an increase in typically rare but potentially pathogenic bacteria, including *Klebsiella pneumoniae* and *Enterococcus faecium* [45]. Administration of broad-spectrum antibiotics in mice decreases type-3 innate lymphoid cells, making them more prone to sepsis [46]. In two major retrospective studies, researchers have reported a close association between dysbiosis and susceptibility to sepsis. It was found that patients with impaired microbiota or higher exposure to antibiotics during hospitalization were more prone to sepsis within 90 days post-discharge. In a cohort study of 10,996 patients, the risk of re-hospitalization for sepsis was more than 70% in people following *Clostridium difficile* infection compared to other infections [47]. Prophylactic administration of antibiotics in a murine model of cecal ligation and puncture (CLP)-induced sepsis (a standard method to induce sepsis in rodents) has shown significant prevention of lung damage [48]. Other than their therapeutic actions, antibiotics also affect the gut microbiome. Administration of metronidazole increases the diversity of *Enterococcus* while decreasing *Bacteroidetes* in comparison to other antibiotics [49]. Frequent use of third generation cephalosporins (ceftriaxone) has been shown to increase the colonization of AmpC (beta-lactamases) overproducing *Enterobacteriaceae* along with a smaller increase in multidrug-resistant Gram-positive bacteria [50]. Clindamycin, which is predominantly excreted through bile, accumulates in feces and significantly disrupts the gut microbiota, resulting in decreasing anaerobes and increasing *Enterobacteriaceae* and Gram-positive bacteria. In addition, it also promotes dysbiosis, which supports the growth of multidrug-resistant pathogens and elevates the risk of developing *Clostridium difficile* colitis [51]. Administration of amoxicillin alone or with beta-lactamase inhibitor alters the gut microbiome and is linked to a decrease in *Lactobacillus* spp. and an increase in *Enterobacteriaceae* [52]. It has been reported that a predominance of *Enterococcus* in the gut is correlated with a higher risk of death in septic intensive care unit (ICU) patients. Although the direct relationship between septic mortality rates and the dominance of *Enterococcus* remains unknown, it can lead to several changes [53]. These shifts may result in decreased SCFA levels, increased virulence, and antimicrobial resistance [39,54]. Following antibiotic use, the restoration of the microbiota may require weeks, months, or even years. Nutritional deprivation in septic patients is associated with an increased use of antibiotics and more severe gut dysbiosis, with reduced SCFAs and anaerobes and a predominance of pathogens. All these factors are linked to bacteremia, organ dysfunction, and higher risk of mortality [55]. While antibiotics are essential to eradicate infection, their overuse can exacerbate sepsis and contribute to antibacterial-resistant bacteria. Antibiotic stewardship programs which encourage the judicious use of antibiotics, helping to maintain a healthy intestinal flora and mitigate the risk of antibiotic resistance, have been implemented in many healthcare settings [56]. Furthermore, probiotics, prebiotics, and synbiotics should be used in combination with antibiotics to restore a healthy gut flora.

## 5. Mechanisms of Liver Injury During Sepsis

**Figure 1:**
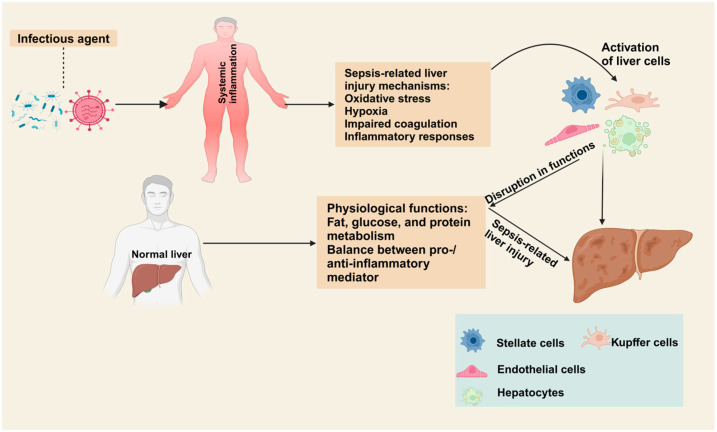
The role of the liver in health and disease. This figure summarizes the physiological functions of the liver and the disruption of its functions during sepsis.

The liver is a vital organ that plays many physiological roles, including detoxification, fat and glucose metabolism, coagulation, and protein biosynthesis [57]. It has several immune and parenchymal cells which contribute to its immunological functions. After the gut barrier, the liver acts as a secondary immune defense through which microbes and their by-products are transported via the portal vein [58]. The balance of pro- and anti-inflammatory molecules and pathways is essential for sustaining immune stability in normal body conditions. An imbalance between pro- and anti-inflammatory molecules can provoke a widespread immune response, potentially leading to organ dysfunction orchestrated by parenchymal, non-parenchymal, and immune cells within the liver [59]. During sepsis, the liver plays a pivotal role in mediating inflammatory responses, coagulation, and clearing pathogens and toxins from the body. These functions help in the regulation of various complications, such as renal failure, lung injury, and coagulopathy. The immune response of the liver to sepsis acts as a double-edged sword: it is beneficial in fighting against the infection, but it can also lead to negative consequences, including immunosuppression, inflammation, and organ damage [2]. The liver is composed of different types of cells, including hepatocytes (80% of liver parenchyma), Kupffer cells (resident liver macrophages), liver sinusoidal epithelial cells (LSECs), and hepatic stellate cells (HSCs). To protect against pathogen invasion, hepatocytes respond to pro-inflammatory cytokines by producing opsonin and complement factors. Furthermore, hepatocytes also regulate iron homeostasis in a manner that limits bacterial growth [60]. Kupffer cells are responsible for tissue homeostasis, antigen presentation, and pathogen endocytosis. Activation of these cells results in the release of several pro-inflammatory cytokines (IL-1β, IL-6 and TNF-α), chemokines (MIP-2 and MCP-1), reactive oxygen species (ROS), eicosanoids, and nitrogen radicals. These mediators play an important role in the recruitment of neutrophils and monocytes from the bloodstream to the liver to combat pathogen invasion [61]. Hyperactivation of these cellular responses results in the uncontrolled activation of pro-inflammatory mediators (cytokine storm). This activation leads to systemic inflammatory responses and, ultimately, MODS [62]. LSECs are specialized fenestrated cells that line the capillaries of the liver (called sinusoids). These cells have large pore sizes, ranging from 50 nm to 150 nm, and make sieve plates which facilitate the nutrient exchange between the bloodstream and liver parenchyma [63]. Upon exposure to pathogens, Kupffer cells and LSECs act as a primary line of defense by expressing several scavenger receptors and recognition receptors, such as Toll-like receptor-4 (TLR-4) (20, 21). HSCs are perisinusoidal cells present in the liver architecture and act as antigen-presenting cells activating NK cells. HSC activation can also affect pro-inflammatory cytokines such as IL-4, IL-17, and TNF-α, which can promote inflammation [64]. Increased oxidative stress is another important factor in the development of sepsis-associated liver injury. Lipid production activated by oxygen radicals results in the production of higher levels of reactive nitrogen and oxygen species. This mechanism significantly damages mitochondrial activity in hepatocytes and induces cell death through apoptosis or necrosis, potentially causing liver injury or failure [65]. Hepatic microcirculatory thrombotic complications also play an important role in aggravating sepsis-induced liver injury. In sepsis, LSECs increase the activation and expression of adhesion molecules. This overexpression results in the deposition of fibrin in the hepatic microvasculature. This impairment decreases the hemodynamic flow and causes hepatic ischemia with thrombosis and the depletion of coagulation factors. The initiation of this cascade can contribute to impaired clotting function, disseminated intravascular coagulation, and death [66]. In animal models, sepsis-related pathological changes in liver injury have been widely studied. In CLP-induced sepsis in rats, physiological functions such as detoxification and glucose and protein metabolism were suppressed, alongside increased levels of unconjugated bile acids [67]. During sepsis, the liver is infiltrated by many inflammatory cells, which exacerbate the inflammatory responses and result in hepatic injury. The physiological functions of the liver and the disruption of its functions during sepsis are summarized in Figure 1. 

## 6. Gut Barrier Function in Sepsis

The permeability of the intestinal barrier plays several important functions, such as the absorption of essential nutrients, inhibiting the transfer of toxic substances from the gut to the bloodstream, and regulation of microbiota–host interactions [68]. Inflammation plays a crucial role in the progression of intestinal damage during sepsis. Sepsis alters the expression and regulation of tight-junction proteins such as zonula occludens-1, claudins, occluding, and junctional adhesion molecules. Meanwhile, myosin light-chain kinase (MLCK), an enzyme important for the regulation of the contraction of smooth muscles, is activated, altering intestinal permeability [69,70]. Sepsis contributes directly to intestinal stem cell proliferation and apoptosis through the activation of TLR4 by PAMPs such as lipopolysaccharides (LPSs) [71]. Dysregulated apoptosis of intestinal epithelial cells (IECs) also affects gut barrier permeability. This gut hyperpermeability leads to changes in the mucus layer, including thickening, impaired adhesion, villous atrophy, and decreased lumen coverage [72,73]. It has been reported that Bcl-2 (an anti-apoptotic protein) overexpression mitigates sepsis-induced hyperpermeability by altering tight junctions in a mouse model of CLP-induced sepsis [74]. Another important factor in the modulation of the gut barrier and intestinal hyperpermeability is the production of pro-inflammatory mediators, including IL-6, TNF-α, MCP-1, and MIP-2 [75,76]. Elevated levels of pro-inflammatory mediators are mainly associated with MLCK contributing to increased gut permeability [77]. It has been shown that in CLP-induced sepsis, MLCK knockout in mice prevented gut hyperpermeability, which was associated with better survival rates [78]. Translocation of bacteria and toxins also contributes to the activation of systemic inflammation and the progression of organ dysfunction and, eventually, failure [79]. Following gut barrier dysfunction, gut microbiota-derived by-products may be transported to other organs through circulation and lymphatic vessels [80]. In surgical patients, it has been found that translocation of bacteria significantly increases the risk of postoperative sepsis [81]. Furthermore, it has been observed that the diversity of gut microbiota decreases in severely ill septic patients, with its composition being increasingly dominated by multidrug-resistant (MDR) organisms [82]. Treatment with broad-spectrum antibiotics (such as cephalosporins, carbapenems, and fluoroquinolones) can also modulate intestinal microbiota in critically ill patients [41,83]. The underlying mechanisms involved in sepsis-related barrier dysfunction are intricate and not completely understood. To clearly elucidate these molecular mechanisms, more research is needed.

## 7. Role of the “Gut–Liver Axis” in Sepsis

The intricate connection between the gastrointestinal and hepatic systems, facilitated by the portal vein, is a fascinating aspect of the gut–liver axis [84]. This axis plays a pivotal role in the host’s responses to sepsis [85]. Upon exposure to pathogens or other stimuli, the liver prevents and mitigates the effects of systemic infections by clearing pathogens from circulation and producing inflammatory mediators [86]. The human gut harbors approximately 100 trillion microbes and serves as a reservoir of over 1 g of LPSs, which are present in the plasma of healthy individuals [87]. The passage of LPS molecules through the small intestine depends on their size and occurs through several mechanisms. These pathways include clathrin-dependent endocytosis, the chylomicron pathway, lipid raft-dependent endocytosis, goblet cell-mediated antigen passages, the paracellular pathway, and micropinocytosis [88,89]. Transportation of LPSs within the colon is dependent on clathrin-mediated or vesicular protein transport pathways [90]. Under physiological conditions, these mechanisms in the intestinal barrier prevent the migration of larger LPS molecules into the bloodstream from the gut. During sepsis, inflammation and dysregulated IEC apoptosis affect these mechanisms and facilitate the transfer of LPSs to distant organs, provoking an overwhelming immune response [91,92]. Once intestinal LPSs enter the bloodstream via the portal vein, the liver metabolizes and detoxifies them. Scavenger receptor (SR)-mediated phagocytosis in the liver takes up LPSs, which then undergo detoxification through the actions of acylhydrolase (AOAH) and alkaline phosphatase [93,94]. AOAH contributes significantly to controlling LPS toxicity by selectively eliminating secondary fatty acyl chains linked to the primary chain in the lipid A fraction [95]. Several plasma proteins, such as CD14, lipoproteins, LPS-binding protein (LBP), and bactericidal/permeability-increasing protein (BPI), also play an important role in LPS detoxification. In the acute stage of sepsis, hepatocytes and IECs predominantly synthesize LBP in mice [96], which recognizes and attaches to the lipid A part of LPS and forms the LPS-LBP complex. This complex promotes the migration and binding of LPSs to membrane CD14 on the surface of neutrophils, monocytes, and macrophages. TLR4 and myeloid differentiation-2 recognize the CD14-LPS complex and initiate an inflammatory response [97]. Activation of TLR4 signaling can cause a significant release of cytokines and cell damage. On the other hand, increased levels of LBP and soluble CD14 in plasma can reduce the severity of systemic responses to LPSs [98]. Gut bacteria also metabolize bile acids produced by the liver and ameliorate host metabolism and immune responses [99]. The liver also secretes bile acids into the small intestine, which plays an important role in shaping the composition of gut microbiota [100]. The liver produces bile acids from cholesterol which are further metabolized by gut microbes in the distal part of the small intestine and colon [101]. During hepatic dysfunction, elevated levels of bile acids provoke inflammation and oxidative stress responses, leading to cirrhosis, apoptosis, fibrosis, and necrosis [102,103]. Bile acids also act as important signaling molecules [104]. Bile acids exert their regulatory effects via G protein-coupled receptors, including Takeda G protein-coupled receptor 5 (TGRP) and farnesoid X receptor (FXR) [105]. The gut microbiota modulate receptor signaling by metabolizing bile acids [106]. The activation of FXR by bile acids in the intestine stimulates the expression of fibroblast growth factor 15 (FGF15). FGF15, in turn, reduces the levels of bile acids by downregulating cholesterol 7α-hydroxylase (CYP7A1) in the liver through a gut microbiota–liver feedback mechanism [101]. Bile acids also exhibit bactericidal properties and help in the maintenance of intestinal barrier integrity by decreasing the permeability of endotoxins, whereas an increased deconjugation of bile acids affects the bactericidal activities of bile and promotes the growth of pathogenic bacteria. This leads to an increased deconjugation of bile acids, which in turn causes bacterial translocation into the bloodstream, resulting in endotoxemia [107]. In summary, alterations in bile acid metabolism by intestinal microbiota contribute significantly to the pathogenesis of sepsis-associated liver injury. Disruptions in the gut–liver axis caused by changes in bile acid signaling result in microbial imbalance and liver inflammation [108]. This intricate relationship between the gut microbiota, liver, and bile acids can exacerbate organ inflammation and injury in sepsis. Crosstalk between liver function and microbial activity serves as a vital defense mechanism through the activation of the immune system against pathogens. Kupffer cells protect against blood-borne pathogens and maintain a sterile circulatory system [109], eliminating microbial by-products through macrophage complement receptor immunoglobulin (CRIg)-mediated pathways [110]. However, CRIg not only helps in the clearance of microbiome-derived products but also recognizes and attaches to lipoteichoic acid (a virulence factor for Gram-positive bacteria) to capture the circulating bacteria [111]. D-lactate (a metabolite produced by commensal bacteria) enters the liver via the portal vein and stimulates Kupffer cells to trap and eliminate pathogens from the bloodstream [112]. This clearance of pathogens from circulation is important to control the spread of pathogens to other organs during infection. A substantial body of evidence suggests that it is important to maintain an association between the gut and liver to control fatal conditions such as sepsis.

## 8. Role of Inflammatory Mediators—Cytokines, Chemokines, Adhesion Molecules, H_2_S, and SP—In Sepsis-Associated Liver Injury

In sepsis, the delicate balance between pro- and anti-inflammatory responses is a crucial factor. The interplay of cytokines, chemokines, adhesion molecules, H_2_S, and SP is a complex and challenging aspect of sepsis-associated liver injury. During sepsis, a network of immune cells, including Kupffer cells, myeloid-derived suppressor cells (MDSCs), NKCs, antigen-presenting cells (APCs), T helper cells (CD4+), and cytotoxic T cells (CD8+), engages in complex communication. This intricate interaction is crucial in maintaining the delicate balance between local and systemic inflammatory responses [2]. Notably, in the early phases of sepsis, Kupffer cells increase the activation of many pro-inflammatory regulators such as MCP-1, MIP-2, IL-6, IFN-γ, and TNF-α [113]. A study by our group has revealed that the inactivation of Kupffer cells by gadolinium chloride (GdCl_3_) significantly reduces liver injury in CLP-induced sepsis in mice. This is associated with decreased levels of liver enzymes (alanine transaminase (ALT) and aspartate aminotransferase (AST)), LSEC defenestration, and attenuation of the activation of pro-inflammatory mediators (TNF-α, IL-6, MCP-1, and MIP-2) [114]. The activation of Kupffer cells also affects the expression of adhesion molecules such as intercellular adhesion molecule 1 (ICAM-1) and vascular cell adhesion molecule 1 (VCAM-1). It has been found that upon stimulation with LPS, Kupffer cells secrete TNF-α, which significantly upregulates the expression of ICAM-1 on LSECs. The administration of dexamethasone, on the other hand, suppresses the release of TNF-α in Kupffer cells stimulated by LPS. Consequently, it downregulates ICAM-1 expression and decreases neutrophil adhesion on LSECs [61]. Recently, we have shown that Kupffer cell inactivation changes the expression of endothelial adhesion molecules in CLP-induced sepsis. Kupffer cell inactivation downregulates the expression of ICAM-1 in the liver but upregulates ICAM-1 expression in the lung [115]. Modulation of Kupffer cell activity could be a novel therapeutic approach to ameliorate sepsis-induced liver injuries, and H_2_S also plays an important role in sepsis-induced liver injury. Enzymes such as cystathionine γ-lyase (CSE) and cystathionine β-synthetase (CBS), responsible for the production of H_2_S, are abundantly present in the liver. Increases in the expression of CSE in the liver and circulating H_2_S levels are found in the CLP-induced sepsis model. The administration of the CSE inhibitor propargylglycine (PAG) significantly improved survival and decreased sepsis-related liver and lung injury in CLP-induced mice, whereas treatment with the H_2_S donor sodium hydrogen sulfide (NaHS) exacerbated liver and lung injury [116]. In sepsis, H_2_S acts as an inflammatory mediator acting through adhesion molecules, cytokines, and chemokines. Signaling mechanisms such as extracellular signal-regulated protein kinase (ERK) and nuclear factor kappa-light-chain-enhancer of activated B cells (NF-κB) are involved in the pro-inflammatory action of H_2_S [117]. LPS-stimulated RAW264.7 cells showed increased mRNA and protein expression of CSE, chemokines (MCP-1), and cytokines (IL-6, TNF-α, and IL-1β). CSE gene silencing with siRNA, on the other hand, downregulates the expression of chemokines and cytokines via the ERK-NF-κB pathway [118]. SP, another inflammatory mediator, contributes to the progression of sepsis [119]. In a CLP sepsis model, it was found that in mice deficient in the SP gene (*Tac1*), the liver sinusoidal damage caused by sepsis was reduced, highlighting the potential harmful effects of SP on liver injury [120]. CD8+ T cells also contribute to sepsis-related liver injury. In an in vivo study, it was observed that numbers of CD8+ T cells were significantly high in the liver after CLP [121]. CD8+ T cells trigger inflammation by producing cytokines or targeting infected cells. In CD8^−/−^ mice, the levels of IL-6 were decreased in comparison to wild-type. However, when CD8+ T cells from wild-type mice that had undergone CLP were transferred to CD8+ T cell^−/−^ mice, they triggered high IL-6 production and liver damage. In septic mice, CD8+ T cells expressed the Fas ligand, which activated NF-κB and triggered the production of pro-inflammatory cytokines. The increased infiltration of CD4+ and CD8+ T cells in the liver upregulated Fas and FasL (proteins important for apoptosis) and increased apoptosis. Higher CD8+ T cell counts were linked to both inflammation and liver damage in sepsis [122].

Furthermore, it has been shown that phosphatase and tensin homolog deletion on chromosome 10 (PTEN) promotes the AKT-mediated β-catenin signaling pathway in the ischemic liver. This highlights the crucial role of the AKT-mediated β-catenin signaling pathway in regulating hepatic inflammation [123]. It has been reported that PTEN-dependent AKT signaling significantly increased the number of Lgr5+ (leucine-rich repeat-containing G protein-coupled receptor 5) cells, while Wnt/β-catenin pathway inhibition suppressed their proliferation in diethylnitrosamine-induced liver cancer [124,125]. Together, these findings suggest that immune cells, inflammatory mediators, adhesion molecules, H_2_S, and SP play significant roles in sepsis-induced liver injury, highlighting their potential therapeutic targets.

## 9. From Bench to Bedside

### Diagnostic and Prognostic Biomarkers for Sepsis and Their Role as Potential Therapeutic Targets

The current clinical diagnostic criteria for sepsis are primarily focused on the Sequential Organ Failure Assessment (SOFA) score, whereas biomarkers that specifically indicate infection are lacking. Conventional methods such as microbiological culture and identification of pathogens are widely used to diagnose infections, but these methods are time-consuming and show low detection rates. C-reactive protein (CRP), a non-specific marker of inflammation, is routinely used in laboratories around the world to assess inflammation in patients with sepsis [126]. During past decades, more than 200 biomarkers of sepsis such as procalcitonin (PCT), IL-6, H_2_S, and SP have emerged. In response to infection or sepsis, levels of PCT start increasing within two to four hours, and within twenty-four hours, the concentration of PCT rises over the hundreds or even 1000 times [127]. A cohort study of 295 patients has shown that patients with lower PCT levels had a 93.4% survival rate after 28 days. Patients with elevated PCT levels had a 51.7% survival rate after 28 days. These findings indicate that increased levels of PCT are associated with a higher risk of death in sepsis patients [128]. In healthy individuals, IL-6 levels fall between 1 pg/mL to 25 pg/mL; however, levels rise above 1 ng/mL during sepsis [129]. The pro-inflammatory role of H_2_S and SP in animal sepsis models has been well-studied, yet their specific function in human sepsis remains largely unknown [117,130,131]. In CLP-induced sepsis mice, it was observed that the concentration of SP in lung tissue and plasma was significantly elevated, whereas in mice deficient in the SP gene preprotachykinin A (PPTA), lung injury and inflammation were markedly mitigated [132]. A clinical study by our group indicated that serum H_2_S and SP levels were elevated in sepsis patients compared to healthy individuals. Furthermore, elevated serum H_2_S and SP concentrations were correlated with higher levels of PCT and IL-6 [119]. Recently, another clinical study by our group has shown that elevated H_2_S levels are associated with an initial phase of inflammatory response, while elevated SP levels were associated with later phases [133]. While the preclinical studies are promising, a better understanding of these biomarkers in the progression of sepsis would help understand their role as potential therapeutic targets.

## 10. Gut Microbiota-Targeted Therapies

Despite increasing research and growing evidence in the field of sepsis, significant gaps remain in translating these findings into effective therapeutic strategies. Antibiotic administration to ICU patients usually results in a decreased biodiversity of commensal intestinal bacteria and a promotion of the growth of opportunistic pathogens [39,134]. This disruption affects the physiological and immunological functions of vital organs, such as bile production in the liver [135,136]. Even short-duration antibiotic treatment can disrupt the intestinal microbiome, and these effects can persist for months. In contrast, the administration of broad-spectrum antibiotics may result in long-term dysbiosis with unknown effects in severely ill patients [137]. Studies, mostly preclinical and some early-stage clinical research, suggest that therapeutic strategies specifically targeting the intestinal microbiota, such as probiotics, prebiotics, and fecal microbiota transplantation (FMT), may be useful in sepsis management.

### 10.1. Probiotics, Prebiotics, and Synbiotics

Probiotics, as live bacteria supplements, play a crucial role in shaping the intestinal microbiota by promoting the growth of beneficial bacteria [138]. Several bacteria strains, such as *Lactobacillus*, *Pediococcus*, *Saccharomyces cerevisiae*, *Bifidobacteria*, *Lactococcus*, and *Enterococcus*, are considered a source of probiotics. Among these species, *Bifidobacteria* and *Lactobacillus* are widely used [139]. Administration of probiotics may provide benefits through several mechanisms, such as producing antibacterial products, improving gut barrier function, and maintaining intestinal homeostasis. These mechanisms play an important role in inhibiting the colonization of pathogenic bacteria in the gut and improving immune responses [140]. In a CLP-induced rat model, treatment with *Lactobacillus rhamnosus* GG suppressed the activation of NF-κB and reduced the mRNA and protein expression of TNF-α, MCP-1, and IL-1β in the liver [141]. Prebiotics are non-digestible dietary molecules, specifically oligosaccharides [142]. In a murine model of sepsis, the administration of cellulose partially decreased systemic inflammation, contributing to improved survival through microbial diversity [143]. Synbiotics (a combination of pro- and prebiotics) improve the gut barrier by protecting the host against toxins and maintaining the structure of epithelial cells. Thirteen randomized controlled studies consisting of 962 patients showed the administration of probiotics and synbiotics significantly reduced the incidence of postoperative sepsis in patients than the control group. Furthermore, postoperative patients treated with synbiotics also had a shorter duration of antibiotic use [144]. A clinical study consisting of 4556 infants reported that the administration of *Lactobacillus plantarum* ATCC-202195 could prevent the onset of sepsis in infants [145]. These studies provide preliminary evidence that these therapies can serve as potential therapeutic agents in future sepsis treatments and prevention.

### 10.2. Fecal Microbiota Transplantation (FMT)

FMT is the process of administering donor fecal material to the recipient’s gut to modify their microbiome and improve health [146]. In the 1950s, FMT was used for the first time to treat pseudomembranous colitis [147]. In relation to the gut–liver axis, several studies have highlighted the positive effects of FMT on sepsis-induced liver injury [148,149]. In a CLP mouse model, FMT increased SCFA production, improved the composition of the mucosal layer, and decreased gut permeability and the inflammatory response [150]. In the case of lethal sepsis caused by pathogens from a septic patient, it has been found that FMT can prevent death in mice. Its protective effects are linked to an increase in butyrate-producing bacteria, improved elimination of pathogens, and immune responses mediated by interferon regulatory factor 3 [151]. Furthermore, in septic rats, FMT increased the survival rate by stimulating the expression of tight junctions in the gut [152]. While there is a dearth of evidence that this approach would work in the clinic, there is one case report in which it has been reported that FMT significantly reduced the severity of postoperative sepsis and diarrhea. These findings suggest that FMT may provoke immune and microecological responses that maintain gut homeostasis by targeting the gut microbiota. Thus, FMT may serve as a potential target for sepsis management [153]. These preclinical and early-stage clinical studies (Table 1) show the promise of gut microbiota-targeted therapies for sepsis and should be the subject of future investigation.

## 11. Conclusions

**Figure 2:**
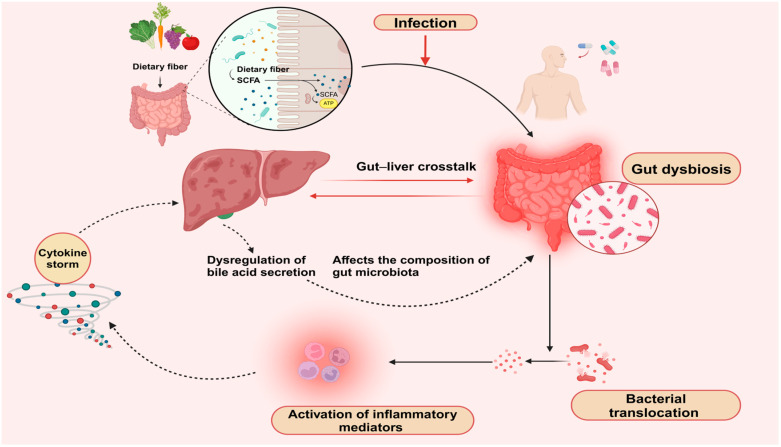
Gut–liver crosstalk and disruption in functions. After exposure to infectious agents, antibiotic administration causes gut dysbiosis and bacterial translocation to the bloodstream. This translocation results in an increased activation of inflammatory mediators, which affects the liver and the composition of the gut microbiota.

Sepsis remains a major cause of morbidity and mortality worldwide, and understanding the gut–liver crosstalk, the disruption of the gut microbiota after antibiotic administration, and its effect on the liver during sepsis is important. These processes are summarized in Figure 2. In this review, we have highlighted the intricate bidirectional communication between the gut and the liver, the interaction of liver cells with inflammatory mediators, and the mechanisms that contribute to sepsis-related liver injury. Additionally, the use of antibiotics influences microbial diversity, further compromising the gut barrier and exacerbating sepsis outcomes. Diagnostic and prognostic biomarkers, such as PCT, IL-6, H_2_S, and SP, provide valuable insights into disease progression and may guide therapeutic interventions. Emerging therapies that target the gut microbiota, including probiotics, prebiotics, synbiotics, and FMT, show promise in restoring microbial balance and improving sepsis treatment. Moving forward, integrating gut microbiota-focused therapies with precise biomarker monitoring could improve outcomes for septic patients, offering a path from the bench to bedside that leverages the gut–liver axis for novel therapeutic approaches.

## Figures and Tables

**Table 1:** Use of gut microbiota-targeted therapies in preclinical and clinical studies.

Type of Study (Preclinical or Clinical)	Probiotics, Prebiotics, Synbiotics, and FMT	Targeted Population	Intervention	Actions	References
Preclinical studies	*Lactobacillus rhamnosus* GG	CLP-induced rat model	Administered before CLP through oral gavage	Reduced mRNA and protein expression of TNF-α, MCP-1, and IL-1β in liver	[141]
	Cellulose	CLP-induced mice model	Administered 2 weeks prior to surgery	Increased abundance of *Akkermansia* and *Lachnospiraceae*; improved survival rate	[143]
	Fecal microbiota transplantation	CLP-induced septic male C57BL/6 mice	Fecal content from healthy C57BL/6 mice was transferred to septic mice through gavage	Increased SCFA and *Lachnospiraceae*; improved composition of mucosal layer; decreased gut permeability and inflammatory response; improved survival	[150]
	Fecal microbiota transplantation	Pathogenic strains were directly inoculated in cecum of male C57BL/6 (8–10 weeks old) mice	Healthy mice’s fecal content was transferred through the intraperitoneal route and enema	Increased levels of butyrate and *Bacteroidetes*; decreased pathogenic biodiversity; improved survival rate	[151]
Clinical studies	*Lactobacillus acidophilus*, *Bifidobacterium infantum*, and *Enterococcus faecium*	Premature neonates	Administered two times per day until discharge	Mitigated chances of late-onset sepsis and infectious diseases	[154]
	Multispecies probiotics	Patients with early onset of sepsis	Administered two times per day for one month	Increased colonization of beneficial bacteria and improved gut function	[155]
	*Lactobacillus reuteri* DSM 17938	Premature neonates	Administered one time per day until discharge	More frequent bowel movements; decreased hospitalization duration; improved growth	[156]
	*Lactobacillus plantarum* with fructo-oligosaccharide	Neonates	Started after 2–4 days of delivery, administered for seven days	Decreased rate of neonatal sepsis	[145]
	*Bifidobacterium breve* and *Lactobacillus casei* with galacto-oligosaccharides	Septic patients on ventilation	Started within three days post-admission	Reduced risk of developing ventilator-associated pneumonia and enteritis; increased SCFA level and colonization of beneficial bacteria	[157]
	Fecal microbiota transplantation	Case report: twenty-nine-year-old woman with high-grade fever, severe diarrhea, and sepsis	Fecal material from a healthy donor was transferred through a nasoduodenal tube	Increased colonization of *Firmicutes* and *Bacteroidetes*; lower fever and defecation; decreased colonization of *Proteobacteria*	[158]

## Data Availability

Not applicable.
